# LncRNA PLAC 2 downregulated miR-21 in non-small cell lung cancer and predicted survival

**DOI:** 10.1186/s12890-019-0931-6

**Published:** 2019-09-10

**Authors:** Huan Xia, Ming Xiu, Jinying Gao, Hongyu Jing

**Affiliations:** 1 Department of Respiratory Medicine, The First Hospital of Jilin University, No. 71 Xinmin Street, Changchun, Jilin 130021 People’s Republic of China; 2 Department of Intensive Care Unit Group One, The First Hospital of Jilin University, Changchun, Jilin 130021 People’s Republic of China

**Keywords:** Non-small cell lung cancer, lncRNA PLAC2, miR-21, Survival

## Abstract

**Background:**

LncRNA PLAC2 has been characterized as a tumor suppressive lncRNA in glioma. We investigated the role of PLAC2 in non-small cell lung cancer (NSCLC).

**Methods:**

A total of 187 NSCLC patients were admitted by The First Hospital of Jilin University from December 2010 to December 2014. All the patients were diagnosed by histopathological approaches. Transient cell transfections, RT-qPCR, invasion, and migration ability measurement, were applied for the experiments.

**Results:**

PLAC2 was down-regulated, while miR-21 was up-regulated in NSCLC tissues compared to non-cancer tissues. Low PLAC2 levels in NSCLC tissues were associated with poor survival of NSCLC patients. PLAC2 and miR-21 were inversely correlated, and PLAC 2 over-expression in NSCLC cells resulted in the down-regulation of miR-21. However, miR-21 over-expression did not significantly affect PLAC2 expression. In addition, PLAC2 over-expression resulted in decreased migration and invasion rates of NSCLC cells. MiR-21 over-expression played the opposite role and attenuated the effects of PLAC2 over-expression.

**Conclusions:**

In conclusion, lncRNA PLAC2 down-regulated miR-21 in NSCLC and inhibited cancer cell migration and invasion.

## Background

Non-small cell lung cancer (NSCLC) is the major subtype of lung cancer, with a high prevalence and mortality rate [1]. Metastasis of neoplasms, which occurs in most NSCLC patients, is the major cause of cancer-related death [2, 3]. Therefore, the overall 5-year survival rate of these patients is quite low [3]. Efforts have been paid to the identification of molecular mechanisms involved in the regulation of cancer cell migration and invasion [4, 5]. However, the pathogenesis of NSCLC is still largely unknown, which limits the development of novel therapeutic approaches to improve the survival of NSCLC patients.

Only a small number of genetic alterations has been identified in NSCLC [6, 7], and the functions of those genes are not sufficiently explained in the complicated mechanism of NSCLC. Studies over the past several decades have shown that long non-coding RNAs (lncRNAs, > 200 nt) are critical factors in diverse biological processes [8]. LncRNAs participate in cancer biology by regulating gene expression [9]. LncRNA-targeted cancer therapies have attracted more and more attentions [10]. More efforts should be paid to the characterization of their functions. Recently it was reported that PLAC2, a novel lncRNA, inhibited cancer cell cycle progression in glioma [11], suggesting its tumor suppressive role. The present study was carried out to investigate the role of PLAC2 in NSCLC.

## Methods

### Patients

A total of 187 NSCLC (adenocarcinoma) patients were admitted by The First Hospital of Jilin University from December 2010 to December 2014. All the patients were diagnosed by histopathological approaches. From those patients, our study included 70 cases (all adenocarcinoma, 42 males and 28 females, 41 to 68 years, 53.3 ± 7.3 years) according to strict criteria. Inclusion criteria: 1) newly diagnosed cases; 2) no severe complications; 3) with expected survival time > 3 months; 4) no therapies were initiated. Exclusion criteria: 1) recurrent NSCLC; 2) therapies were carried out before this study; 3) other obvious clinical disorders were observed. There were 15, 15, 23, and 17 cases at AJCC stage I-IV, respectively. Among these patients, non-metastasis (NM) was found in 17 cases, lymph node metastasis-only (LNM) was found in 38 cases, and distant metastasis (DM) was found in 17 cases. All the 70 patients were informed with the experimental principle. Ethics Committee of the hospital above approved this study.

### NSCLC and non-cancer tissues

To detect differential gene expression in NSCLC, adjacent non-tumor, and NSCLC tissues were obtained from each patient during the histological biopsy. Weight of biopsy ranged from 0.07 to 0.13 g. All NSCLC and non-tumor tissues were confirmed by histopathological examinations.

### Follow-up

From the day of admission, our patients were followed up for five years or until their death through telephone or outpatient visit. Patients’ survival conditions were recorded. The ones lost during follow-up, and the ones died of other diseases or accidence were excluded.

### Cell lines

H650 and H1581 (ATCC, USA) human NSCLC cell lines were used. A mixture containing 5% FBS and 85% RPMI-1640 Medium was used as cell culture medium. Cells were cultivated under conditions of 37 °C and 5% CO_2_.

### Transient cell transfections

PLAC2 expression vector was constructed using the pcDNA3 vector by Sangon (Shanghai, China). H650 and H1581 cells were collected at 70–90% confluence and 10 nM PLAC2 expression vector or empty vector (negative control, NC) was transfected into 10^5^ cells using lipofectamine 2000 reagent (Sigma-Aldrich, USA). Negative control miRNA and miR-21 mimic were obtained from Sigma-Aldrich (USA). Through the same way mentioned, 45 nM miR-21 mimic or negative control miRNA (negative control, NC) was transfected into 10^5^ cells. In this experiment. Cells without transfections were regarded as control cells (Control, C). All subsequent experiments were performed using cells harvested at 24 h after transfections.

### RT-qPCR

Extraction of total RNA from tissues and cells was performed using Ribozol reagent (Invitrogen, USA). AMV Reverse Transcriptase XL (Clontech, USA) and QuantiTect SYBR Green PCR Kit (Qiagen, Shanghai, China) were used to prepare reverse transcription and qPCR reaction, respectively. With 18S rRNA as endogenous control, the expression level of PLAC2 was normalized. With GAPDH as endogenous control, PTEN expression level (a target of miR-21) was normalized.

To detect miRNA-21, mirVana miRNA Isolation Kit (Thermo Fisher Scientific), qScript microRNA cDNA Synthesis Kit (Quantabio, USA) and miScript SYBR Green PCR Kit (QIAGEN, Germany) were used to perform miRNA extractions, miRNA reverse transcriptions and qPCR reactions, respectively. U6 was used as the endogenous control of miRNA-21. Three replicates were set for each experiment, and 2^-ΔΔCT^ method was used to process data.

### Invasion and migration ability measurement

H650 and H1581 were harvested at 24 h post-transfections. RPMI-1640 Medium (1% FBS) was mixed with cells at a ratio of 1 ml per 3 × 10^4^ cells to prepare single cell suspensions. Transwell chambers were used to test cell invasion and migration abilities. Matrigel (356,234, Millipore, USA) was used to coat the upper chamber before invasion assay but not migration assay. The upper chamber was added with 0.1 ml cell suspension, and the lower chamber was filled with RPMI-1640 Medium (20% FBS). After cell culture under the conditions of 5% CO_2_ and 37 °C for 10 h, 0.5% crystal violet (Sigma-Aldrich, USA) staining was performed for 16 min at 22 °C. Cells were counted under an optical microscope.

### Statistical process

Mean values in the present paper represented the data from three biological replicates. Paired t-test was performed to test the null hypothesis that no differences exist between two types of tissues. One-way ANOVA was used to test the null hypothesis that no differences exist among multiple groups. If this null hypothesis is false, Tukey test was performed to detect any significant differences. Correlations were analyzed by linear regression. Survival analysis was performed using K-M method and log-rank test. *P* < 0.05 was statistically significant.

## Results

### Expression patterns of PLAC2 and miR-21 were opposite in NSCLC

**Fig. 1:**
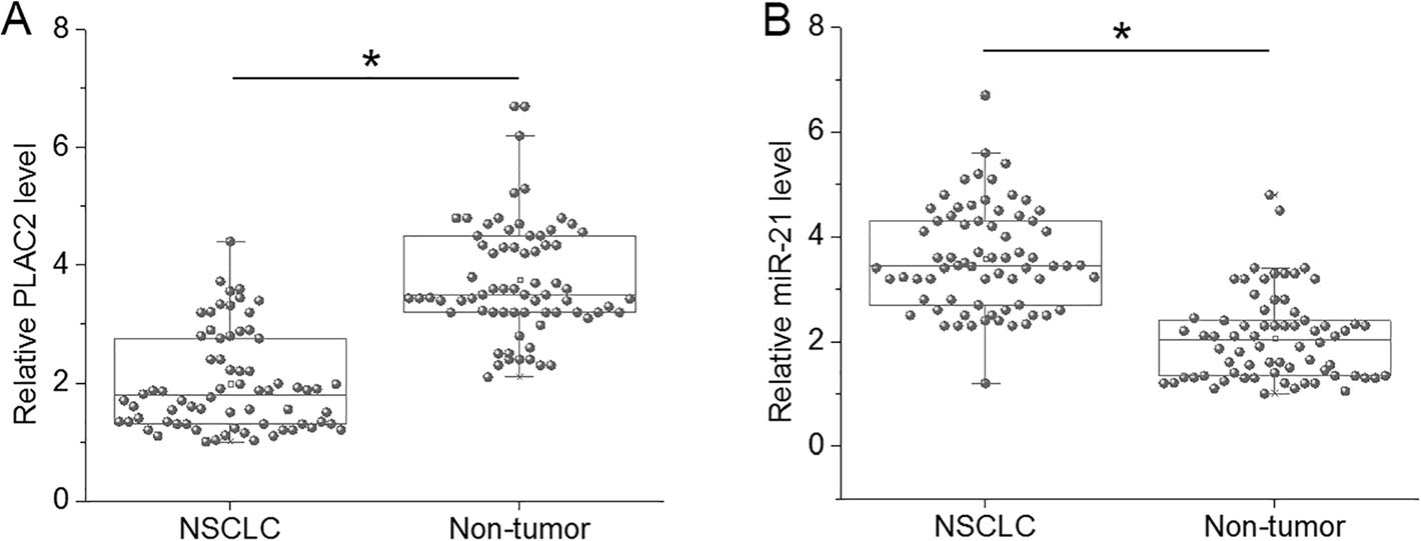
Expression patterns of PLAC2 and miR-21 were opposite in NSCLC. This figure shows the comparison of PLAC2 (**a**) and miR-21 (**b**) expression levels between non-tumor and NSCLC tissues. Expression were detected by performing RT-qPCR and data were analyzed by paired t test (*, *p* < 0.05)

PLAC2 and miR-21 in two types of tissues were detected by performing RT-qPCR, and expression data were analyzed by performed paired t-test. It was observed that compared to the levels in non-tumor tissues, PLAC2 levels were significantly lower (Fig. 1a, *p* < 0.05) and expression levels of miR-21 were significantly higher (Fig. 1a, *p* < 0.05) in NSCLC tissues.

### Low PLAC2 levels predicted poor survival

**Fig. 2:**
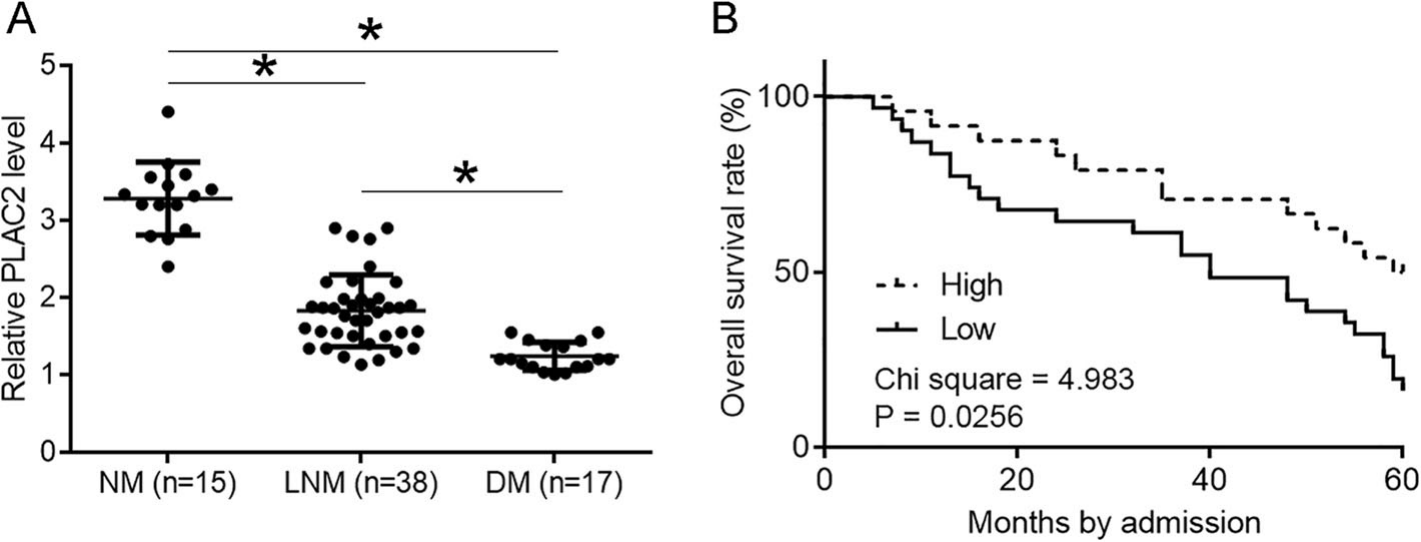
Low PLAC2 levels predicted poor survival. Among 70 NSCLC patients, non-metastasis (NM) was found in 17 cases, lymph node metastasis-only (LNM) was found in 38 cases and distant metastasis (DM) was found in 17 cases. Expression levels of PLAC2 were compared among groups by performing one-way ANOVA and Tukey test. It was found that expression levels of PLAC2 were significantly lower in DM group than in LNM and NM groups. In addition, expression levels of PLAC2 were also significantly lower in LNM than in NM groups (**a**), (*, *p* < 0.05). Survival analysis was performed using K-M method and log-rank test. It was observed that patients with low PLAC2 levels in NSCLC tissues had much worse survival (**b**)

Among 70 NSCLC patients, non-metastasis (NM) was found in 17 cases, lymph node metastasis-only (LNM) was found in 38 cases, and distant metastasis (DM) was found in 17 cases. It was found that expression levels of PLAC2 were significantly lower in the DM group than in LNM and NM groups. In addition, expression levels of PLAC2 were also significantly lower in LNM than in NM groups (Fig. 2a, *p* < 0.05). According to the expression of PLAC2 in NSCLC tissues, 70 patients were divided into high (*n* = 32) and low (*n* = 38) groups (Youden’s index). K-M method was used to plot survival curves, which were compared by performing a log-rank test. The data showed that patients with low PLAC2 levels in NSCLC tissues had much worse survival rate (Fig. 2b).

### PLAC2 over-expression resulted in miR-21 down-regulation in NSCLC cells

**Fig. 3:**
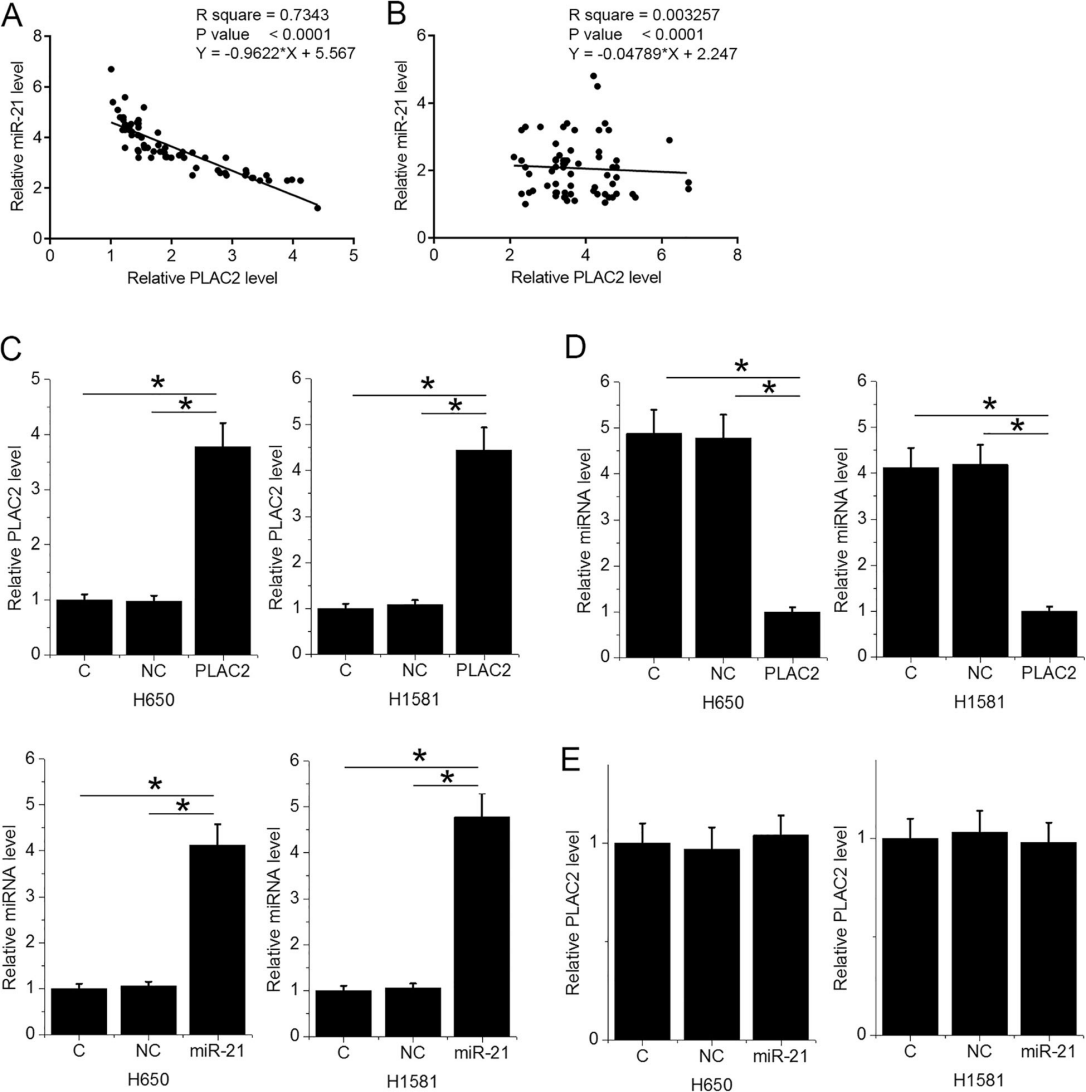
PLAC2 over-expression resulted in miR-21 down-regulation in NSCLC cells. Linear regression was used to analyze the correlation between PLAC2 and miR-21. In NSCLC tissues, PLAC2 and miR-21 were inversely and significantly correlated (**a**). In non-tumor tissues, PLAC2 and miR-21 were not significantly correlated (**b**). PLAC2 and miR-21 over-expression was achieved at 24 h after transfection (**c**). Moreover, PLAC2 over-expression in NSCLC cells resulted in the down-regulation of miR-21 (**d**). However, miR-21 over-expression did not significantly affect PLAC2 expression (**e**)

Correlations between PLAC2 and miR-21 were analyzed by linear regression. In NSCLC tissues, PLAC2 and miR-21 were inversely and significantly correlated (Fig. 3a). In non-tumor tissues, PLAC2 and miR-21 were not significantly correlated (Fig. 3b). Comparing to two controls (C and NC), PLAC2 and miR-21 over-expression was achieved at 24 h after transfection (Fig. 3c, *p* < 0.05). Moreover, PLAC2 over-expression in NSCLC cells resulted in the down-regulation of miR-21 (Fig. 3d, *p* < 0.05). However, miR-21 over-expression did not significantly affect PLAC2 expression (Fig. 3e).

### PLAC2 over-expression resulted in PTEN upregulation at mRNA level in NSCLC cells

**Fig. 4:**
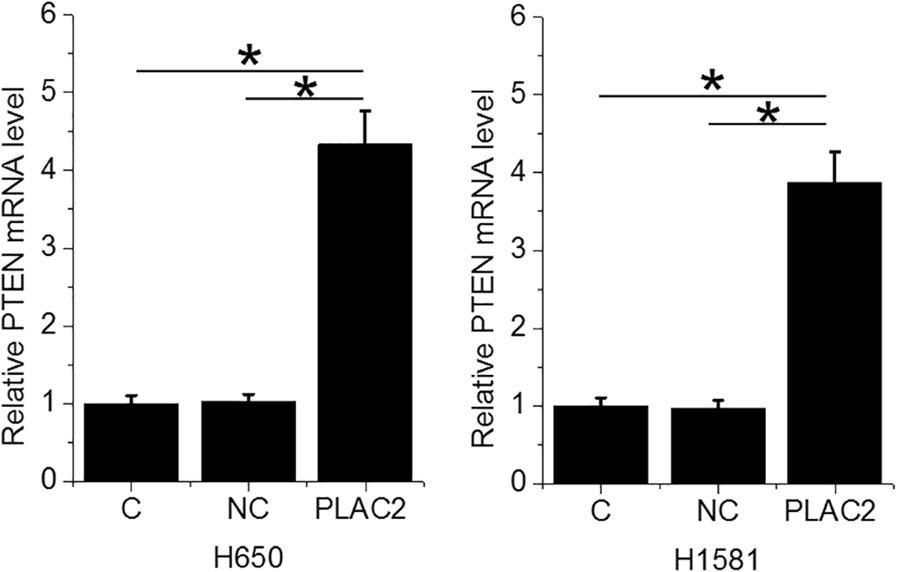
PLAC2 over-expression resulted in PTEN upregulation at the mRNA level in NSCLC cells. PTEN mRNA was detected at 24 h after the transfection of the PLAC2 expression vector, and expression data were analyzed by one-way ANOVA and Turkey test It was observed that PTEN mRNA levels were significantly increased in cells with PLAC2 over-expression (*, *p* < 0.05)

PTEN is a target of miRNA-21. To test the effects of PLAC2 over-expression on PTEN, PTEN mRNA was detected at 24 h after the transfection of the PLAC2 expression vector. It was observed that PTEN mRNA levels were significantly increased in cells with PLAC2 over-expression (Fig. 4, *p* < 0.05).

### PLAC2 regulated NSCLC cell invasion and migration through miR-21

**Fig. 5:**
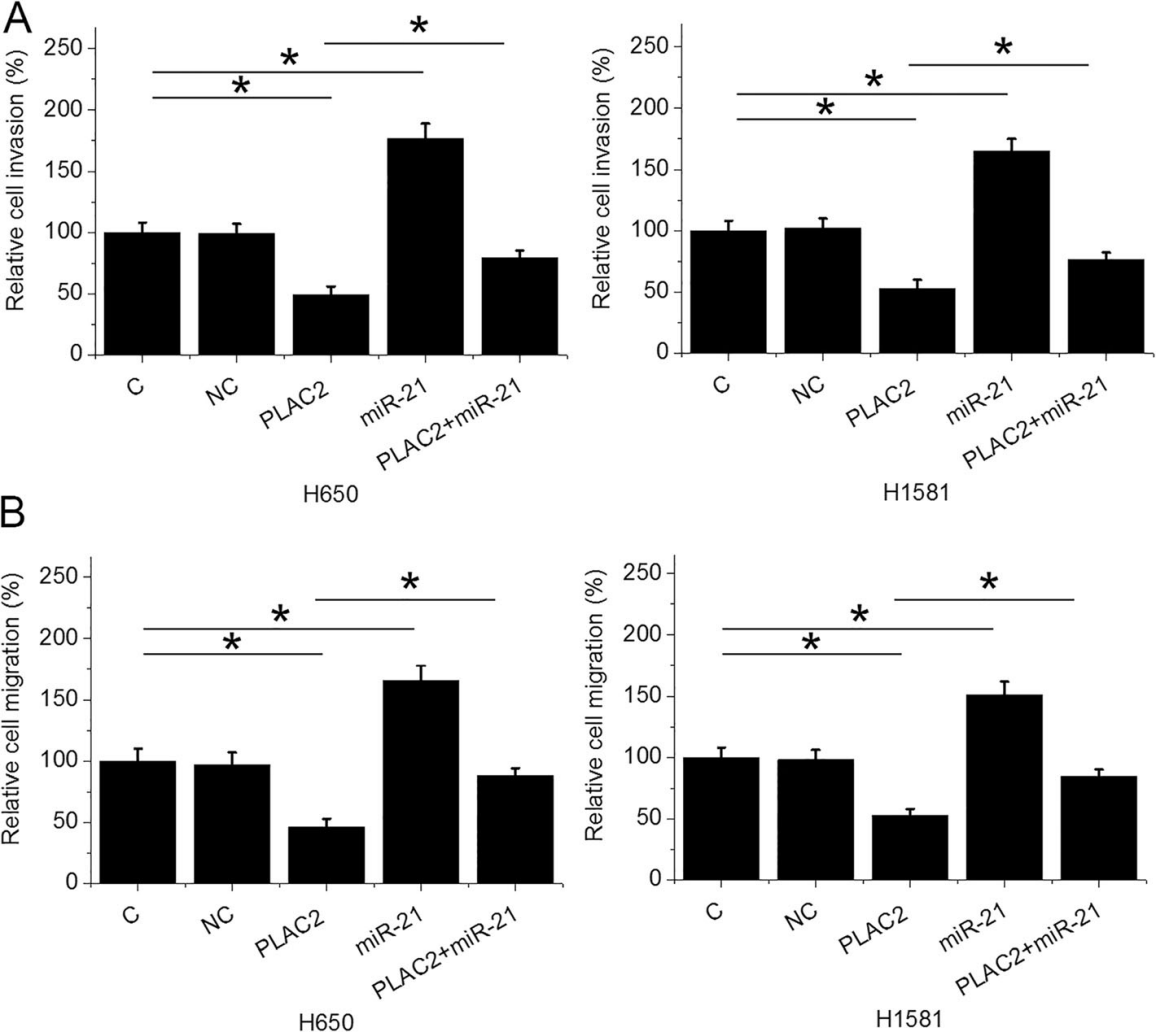
PLAC2 regulated NSCLC cell invasion and migration through miR-21. Cell migration and invasion data analyzed by one-way ANOVA and Tukey test. It showed that compared to two controls (C and NC), PLAC2 over-expression resulted in decreased invasion **a**) and migration (**b**) rates of NSCLC cells. MiR-21 over-expression attenuated the effects of PLAC2 over-expression (*, *p* < 0.05)

Cell migration and invasion data were analyzed by one-way ANOVA and Tukey test. Comparing to two controls (C and NC), PLAC2 over-expression resulted in decreased invasion (Fig. 5a) and migration (Fig. 5b) rates of NSCLC cells (*p* < 0.05). MiR-21 over-expression played the opposite role and attenuated the effects of PLAC2 over-expression (*p* < 0.05).

## Discussion

The expression pattern, function, and clinical potentials of PLAC2 have been investigated in this study. We proved that PLAC2 was a tumor suppressor in NSCLC. PLAC2 over-expression resulted in the inhibited NSCLC cell migration and invasion, possibly through the down-regulation of miR-21.

The function of PLAC2 in inhibiting cancer cell cycle progression has been characterized in glioma [11], while its clinical value has not been evaluated. The prognosis of most NSCLC patients is poor [12, 13], and the accurate prognosis is critical to elongate the survival time through individualized treatment or postoperative care. In this study, we proved that PLAC2 was down-regulated in NSCLC, and the low level of PLAC2 in NSCLC tissues was accompanied by low overall survival rate. Therefore, PLAC2 may be a potential prognostic biomarker for NSCLC. Our future studies will include more patients to further confirm our conclusions.

MiR-21 plays oncogenic roles in almost all types of cancer, and inhibition of miR-21 resulted in suppressed cancer development and progression [14, 15]. Consistently, our study observed the up-regulated miR-21 in NSCLC, as well as increased migration and invasion rates of NSCLC cell after miR-21 over-expression. It is known that miR-21 may be regulated by certain lncRNA in cancer [16]. In this study, we characterized PLAC2 as an upstream inhibitor of miR-21, and the inhibition of miR-21 was involved in the regulation of NSCLC cell migration and invasion. However, miR-21 does not directly affect cell behaviors. Instead, miR-21 acts on downstream cancer-related pathways. For instance, miR-21 directly targets tumor suppressive PTEN, SESN1, CAB39L, and RASA1 in different types of cancer to regulate different cancer cell behaviors [17–19]. In the present study, up-regulated PTEN was observed in NSCLC cells after PLAC2 over-expression. Therefore, PLAC2 may rescue PTEN from the targeting of miR-21, and inhibite NSCLC cell migration and invasion. Our future studies will investigate the involvement of other targets of miR-21 in PLAC2-regulated cancer cell behaviors.

## Conclusions

In conclusion, PLAC2 was down-regulated in NSCLC, and PLAC2 over-expression resulted in inhibited cancer cell migration and invasion by down-regulating miR-21.

## Data Availability

The datasets used and analyzed during the current study are available from the corresponding author on reasonable request.

## Abbreviations

FBS: Fetal bovine serum
lncRNAs: Long non-coding RNAs
NSCLC: Non-small cell lung cancer
